# Ten-Year Minimum Follow-up Study of First Metatarsophalangeal Joint Fusion in Young vs Old Patients

**DOI:** 10.1177/10711007231205567

**Published:** 2023-12-30

**Authors:** Fabrice Scheurer, Stefan M. Zimmermann, Philipp Fischer, Stephan H. Wirth, Silvan Beeler, Arnd F. Viehöfer

**Affiliations:** 1 Balgrist University Hospital, Zurich, Switzerland

**Keywords:** First metatarsophalangeal joint fusion, young, arthrodesis

## Abstract

**Background:**

Painful degenerative joint disease (DJD) of the first metatarsophalangeal joint (MTP I), or hallux rigidus, mainly occurs in later stages of life. For end-stage hallux rigidus, MTP I arthrodesis is considered the gold standard. As young and active patients are affected considerably less frequently, it currently remains unclear, whether they benefit to the same extent. We hypothesized that MTP I arthrodesis in younger patients would lead to an inferior outcome with decreased rates of overall with lower rates of patient postoperative pain and function compared to an older cohort.

**Methods:**

All patients aged <50 years who underwent MTP I arthrodesis at our institution between 1995 and 2012 were included in this study. This group was then matched and compared with a group of patients aged >60 years. Minimum follow-up was 10 years. Outcome measures were Tegner activity score (TAS), a “Virtual Tegner activity score” (VTAS), the visual analog scale (VAS), and the Foot Function index (FFI).

**Results:**

Sixty-one MTP I fusions (n = 28 young, n = 33 old) in 46 patients were included in our study at an average of 14 years after surgery. Younger patients experienced significantly more pain relief as reflected by changes in VAS and FFI Pain subscale scores. No difference in functional outcomes was found with change in the FFI function subscale or in the ability to have desired functional outcomes using the ratio of TAS to VTAS. Revision rate did not differ between the two groups apart from hardware removal, which was significantly more likely in the younger group.

**Conclusion:**

In patients below the age of 50 years with end-stage DJD of the first metatarsal joint, MTP I arthrodesis not only yielded highly satisfactory postoperative results at least equal outcome compared to an older cohort of patients aged >60 years at an average 14 years’ follow-up. Based on these findings, we consider first metatarsal joint fusion even for young patients is a valid option to treat end-stage hallux rigidus.

**Level of Evidence:**

Level III, a case-control study.

## Introduction

Hallux rigidus (HR) often affects about 2.5% of people aged >50 years, and the prevalence of hallux rigidus increases with age.^10,21^ It is considered the second most common disease of the first metatarsophalangeal joint after hallux valgus deformity.^21,29,33^ The condition is typically characterized by pain that increases during walking, limited dorsiflexion, and formation of periarticular osteophytes. Causes leading to destruction of the cartilage of the first metatarsophalangeal joint (MTP I joint) include degenerative changes, trauma, deformities, osteochondrosis dissecans, and inflammatory diseases, such as rheumatoid arthritis, seronegative arthritis, and gout.^2,3,31,32^ Several risk factors have been identified, including hypermobility of the first ray, pronation, hallux valgus, interphalangeal hallux valgus, female gender, and metatarsus adductus.^1,14,30^ A traumatic cause is often associated with unilateral hallux rigidus in younger patients.^24^

As a degenerative disease, osteoarthritis of MTP usually affects older patients with an increasing prevalence of symptomatic MTP I osteoarthritis in older patients.^20^

Therefore, the cohort of studies addressing MTP I fusion is usually advanced in age, with a mean age around 60 years. However, MTP I fusion is sometimes even necessary in young patients, with patients down to 20 years of age included in MTP I fusion studies.^19^

Conservative treatment options consist of oral anti-inflammatory drugs, intra-articular corticosteroid injections, lifestyle modifications, taping, and footwear adjustment.^18,26^ Once conservative treatment fails, various surgical procedures can be considered depending on the extent of joint degeneration. For end-stage osteoarthritis, MTP I arthrodesis is often referred to as the gold standard with reliable clinical and radiologic results.^5,8,12,16,21,25^ As MTP I osteoarthritis most commonly affects patients in the sixth decade of life and older, the cohort of most studies is not representative for younger patients.^11^ To our knowledge, no studies have investigated the long-term outcome of first **metatarsophalangeal** (MTP I ) arthrodesis specifically for patients aged <50 years over at least 10 years.

Therefore, the primary aim of this study was to report the long-term clinical and radiologic outcome of patients who received MTP I arthrodesis below the age of 50 years and compare these results to a matched cohort of older patients from the same institution as well as to previously published results in literature.

We hypothesized that MTP I arthrodesis in younger patients would lead to an inferior outcome with lower rates of patient postoperative pain and function compared with older patients.

## Material and Methods

This case-control study was approved by the local research ethics committee (KEK-ZH-Nr. 2019-01985) and all included patients gave their written consent.

All patients who received MTP-1 joint arthrodesis from January 1995 to December 2012 were included in the study. Exclusion criteria were previous operations of the MTP I joint or first ray besides cheilectomy, patients with underlying diseases that limit mobility to a higher degree (e.g. infantile cerebral palsy), age between 50 and 60 years at the time of surgery, incomplete documentation, or the refusal to participate in the study. If both feet were operated, a minimum span of 1 year between both operations was claimed to minimize confounding.

The investigated variables were visual analog scale, FFI, Tegner Activity Scale, Virtual Tegner Activity Scale, hardware removal, radiologic assessment, and type of fixation.^7,22,35^

To analyze the effect of age on the clinical outcome, 2 groups were defined.

Group A consisted of all patients <50 years of age at the time of surgery and group B all patients aged >60 years. With a minimum follow-up of 10 years, we omitted the group between 50 and 60 years to minimize an overlap bias during the postoperative observation period. During the observation period, a total of 298 patients received an MTP I arthrodesis at our hospital. Of those, 36 patients (42 feet) were younger than 50 years at the time of surgery, 3 (3 feet) were excluded because of previous surgeries, and 11 patients (11 feet) did not respond to several attempts to contact them. A remaining 22 patients (28 feet) were included in the young patients’ group (group A). These were matched to patients who had received MTP I arthrodesis at the age of ≥60 years (24 patients, 33 feet; group B).

The patients were matched according to sex, type of fixation, and history of joint-preserving surgery to exclude possible confounders.

**Figure 1.**
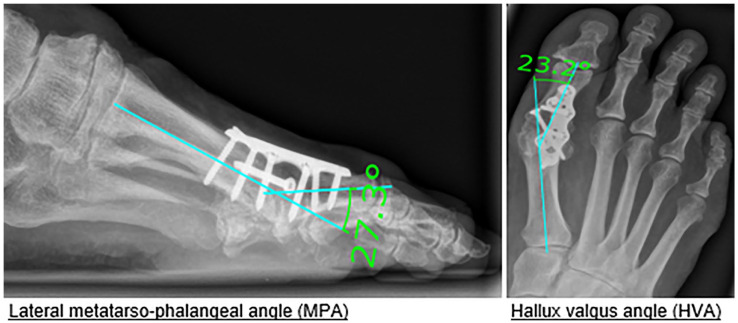
Radiographic assessment.

To identify a potential bias between both groups, a radiographic assessment was performed with an analysis of the 1-year follow up radiograph. For this purpose, the hallux valgus angle (HVA), the lateral metatarsophalangeal angle (MPA), and the pseudarthrosis rate were compared.^9,27,36^ We used the program mediCAD (version V7.0, Hectec GmbH, certified medical device) to measure the angles (Figure 1).

Clinical notes and operative reports were reviewed for patient demographics and the American Society of Anesthesiologists Score (ASA) was recorded.

At final follow-up at least 10 years postoperatively, patients were contacted and patient-specific outcome measures including the visual analog scale (VAS), the FFI, and the Tegner Activity Scale scores were recorded.^4,6,17,28^ To evaluate the desired level of activity, a so called “virtual Tegner Activity Scale” was recorded. Patients could estimate the activity level they would like to have without any physical impairment due to the operated foot using the same score as the Tegner activity level with gradations from 0 to 10.

The rationale was that the TAS cannot be readily compared between young and old patients as the activity level is known to decrease with age. It is a way to answer the question whether the patients are as active as they would like to be or if they perceived physical limitations due to the operated foot. This variable was included in the outcome as a ratio between the TAS and VTAS.

Preoperative scores were analyzed for comparison. If scores were not available, the questionnaires were completed from the electronic medical record where possible.

### Surgical Technique and Postoperative Rehabilitation

MTP I arthrodesis was performed using either a crossed-screw technique or fusion plate arthrodesis. The crossed-screw technique with two lag screws was used in n = 22/61 cases (group A n = 13, group B n = 9). The MTP I fusion plate was used in 39 of 61 cases (group A n = 15, group B n = 24). The postoperative rehabilitation included partial weightbearing for 6 weeks in a lower leg cast in all cases independent of the fixation type.

### Statistics

Confounder-adjusted group comparisons were performed using linear regressions (least squares) with a stepwise inclusion scheme to include potential confounder. Descriptive statistics are provided with mean and SD or as absolute and relative counts. Baseline characteristics were assessed with independent samples *t* tests or Fisher exact tests as applicable. The analysis was conducted with SPSS (version 28.0; IBM Corp, Armonk, NY). *P* values below .05 were considered statistically significant.

## Results

The mean age in group A was 43 (min = 39, max = 49; SD = 5.68) and in group B it was 67 (min = 60, max = 75; SD = 5.80). In group A, 16 (57%) of these were male. In group B, 14 (50%) were male. The mean follow-up in group A was 168 months (min = 121, max = 239; SD = 50.19) and for group B it was 165 months (min = 128, max = 274; SD = 50.31). Demographics and description of both groups are given in Table 1.

**Table 1.** Demographics and Description of the Groups.

Variable	Group A: <50 y	Group B: >60 y	*P* Value*
Patients, n	22	24	
MTP I arthrodesis, n	28	33	
Age, y, mean (SD)	43 (5.68)	67 (5.80)	**.001**
Gender: male, n (%)	16 (57)	14 (50)	.321
Follow-up time, mo, mean (SD)	168 (50.19)	165 (50.31)	.841
Side: right, n (%)	13 (46)	20 (61)	.221
ASA stage, mean (SD)	1.8 (0.52)	2.1 (0.62)	.086
Type of fixation: 2 screws, n (%)	13 (46)	10 (30)	.123
Type of fixation: plate, n (%)	15 (54)	23 (70)	.123
Previous cheilectomy, n (%)	5 (18)	1 (3)	.077
Hardware removal, n (%)	11 (39)	4 (12)	**.015**
Other revisions, n (%)	5 (18)	4 (12)	.483

Abbreviation: MTP I, first metatarsophalangeal.

* Boldface indicates significance (*P* < .01).

We analyzed possible confounders for both groups: In our radiologic assessment at the final follow-up, there was no difference between the 2 groups in hallux valgus angle (HVA), metatarsophalangeal angle (MPA) lateral and the rate of pseudarthrosis (Table 2). The 2 groups did not differ in terms of radiographic malalignment (HVA 0.584, lateral MPA 0.199), or pseudarthrosis rate (*P* = .253).

**Table 2.** Postoperative Radiographic Parameters of the Groups.

Variable	Group A: <50 y	Group B: >60 y	*P* Value
HVA, degrees, mean (SD)	10.4 (6.2)	11.3 (6.5)	.548
Lateral MPA, degrees, mean (SD)	15.9 (6.5)	18.2 (7.2)	.199
Rate of pseudarthrosis, n (%)	1 (4)	0 (0)	.253

Abbreviations: HVA, hallux valgus angle, MPA, metatarsophalangeal angle.

Both groups showed a significant pain reduction (group A from preoperative VAS 4.7 to postoperative 1.0, and group B from preoperative VAS 4.1 to postoperative VAS 1.4), as well as improved clinical outcome (FFI) as can be seen in Table 3.

**Table 3.** Comparison of Postoperative Outcome Parameters

Variable	Preoperative	Postoperative	*P* Value
Group A (n=28)			
VAS (SD)	4.7 (1.27)	1.0 (1.59)	.01
FFI pain (SD)	37.3 (11.14)	11.7 (13.48)	.01
FFI function (SD)	46.1 (11.96)	18.7 (17.61)	.01
Group B (n=33)			
VAS (SD)	4.1 (1.19)	1.4 (1.88)	.01
FFI pain (SD)	33.0 (9.41)	17.0 (14.07)	.01
FFI function (SD)	47.8 (15.54)	28.6 (23.72)	.01

Abbreviations: FFI, Foot Function Index; VAS, visual analog scale.

Young patients showed a significantly higher pain reduction measured by FFI pain (mean 25.6, SD = 15.0, *P* = .014) and VAS score difference (3.7, SD = 1.9, *P* = .048) compared to the older patients with a FFI pain (mean 16.0, SD = 13.2, *P* = .014) and VAS score difference (mean 2.7, SD = 2.0, *P* = .048) at the final 165-month follow-up (SD = 50.3, *P* = .841) following MTP I arthrodesis.

As expected, younger patients were more active, reflected by a higher Tegner score (group A 4.0, SD 1.8; group B 2.9, SD 0.8). Regarding the difference of the Tegner Score to the Virtual Tegner Score, there was no difference between the groups (0.465) (Table 4).

**Table 4.** Group Comparison Preoperation, at the Final Follow-up, and the Difference Between Pre- and Postoperation.

Score	Group A: <50 y, Mean (SD)	Group B: >60 y, Mean (SD)	*P* Value^a^
VAS score			
Preoperation	4.7 (1.3)	4.1 (1.2)	**.043**
Final	1.0 (1.6)	1.4 (1.9)	.295
Difference	3.7 (1.9)	2.7 (2.0)	**.048**
FFI pain			
Preoperation	37.3 (11.1)	33.0 (9.4)	.093
Final	11.7 (13.5)	17.0 (14.1)	.129
Difference	25.6 (15.0)	16.0 (13.2)	**.014**
FFI function			
Preoperation	46.1 (12.0)	47.8 (15.5)	.624
Final	18.7 (17.6)	28.6 (23.7)	.056
Difference	27.4 (17.9)	19.2 (23.9)	.152
Final TAS score	4.0 (1.8)	2.9 (0.8)	**.013**
Final V-TAS score	4.8 (1.6)	3.4 (0.7)	**.001**
TAS to V-TAS difference	0.75 (1.24)	0.51 (0.77	.465

Abbreviations: FFI, Foot Function Index; TAS, Tegner Activity Score; VAS, visual analog scale; V-TAS, Virtual Tegner Activity Score.

a Boldface indicates significance (*P* < .01).

### Revision

The overall revision rate for hardware removal alone was 25% (n = 15/61); in group A (n = 11) 39% and in group B (n = 4) 12% (*P* = .015). Further revisions (n = 9/61) included 3 pseudarthrosis revisions in each group (group A = 11%, group B = 10%), 1 revision for disturbing hyperkeratosis in group B (3%), 1 revision for screw malposition in group A (4%), and finally 1 revision for MTP I arthrodesis consolidated in dorsiflexion in group B (3%).

Comparing the 2 groups, the young group required hardware removal significantly more often (*P* = .015). There were more previous cheilectomies in group A, although this difference did not reach statistical significance (*P* = .077). There were no significant differences in fixation type (*P* = .123) and all other revisions (*P* = .483) between the 2 groups.

Specifically, we tested whether gender, type of fixation, cheilectomy, side of surgery, revision rate, or hardware removal had an independent impact on the outcome variables. Independent samples *t* tests were performed to test whether outcome parameters differed as a function of certain baseline variables (Supplemental Table 1).

We noticed a trend for patients with a higher lateral MPA toward an inferior function. However, there was no statistically significant correlation regarding the scores at the final follow-up (FFI pain, *P* = .182; FFI function, *P* = .151; and VAS, *P* = .789).

The method of plate fixation was associated with significantly worse function (*P* = .041) as well as smaller improvement in function (*P* = .043). In addition, patients who did not require revision had a higher activity level as measured by the Tegner score (*P* = .039). Moreover, hardware removal had a significant effect on the change in subjective pain perception (*P* = .018). Therefore, these baseline variables are potential confounding variables in the group comparison.

## Discussion

The present study reports a highly satisfactory clinical outcome following MTP arthrodesis for young as well as old patients. As most MTP I joint fusions are performed around or above the age of 60 years, and as both decreased mobility and an increase in comorbidities have been reported in literature around this age, this cut-off was chosen as a standard cohort for comparison with the group of young patients.^15,34^

Contrary to our expectations, young patients showed an even higher pain reduction after MTP I arthrodesis compared to older patients in relation to the FFI (pain) (*P* = .014). This is also reflected in the VAS (*P* = .048), although the value was already significantly different preoperatively (*P* = .043). Regarding clinical outcome in long-term follow-up, measured by FFI (function) (0.158), there was a slight difference in favor of the young group, but not significant. Likewise, the younger patients had the same gain of function compared to the older patients.

In a quite similar study with a much shorter follow-up (1 year postoperatively), no difference in pain and function was found between young and old patients.^23^ The main difference for this discrepancy with our study we see in the age cutoff, with young patients defined as age ≤65 years, for us up to 50 years, but also the follow-up time and the other clinical scores might have had an influence (ie, 36-Item Short Form Health Survey [SF-36] and Life-Space Assessment survey [LSA]). Another study showed that in young patients (mean age 49 years), 96% (n = 48/50) were satisfied with their level of sports activity after 1-2 years following MTP I arthrodesis.^13^ Again, we see the biggest difference in the shorter follow-up time and the other clinical scores used (eg, Foot and Ankle Outcome Score [FAOS], Activities of Daily Living [ADL], and Quality of Life [QoL]).

As the Tegner score inversely correlates with age, it was expected that younger patients showed a higher Tegner activity level.^4^ Interestingly, the difference to the virtual activity level was not significantly increased in the young patient group compared to the older patient group. This indicates that the younger patients, despite having received MTP I arthrodesis, did not have to accept higher physical limitations in their daily life compared with older patients. Based on these findings, potential concerns of younger patients of having to accept a loss of function due to surgery seems unwarranted.

Apart from a significantly higher rate of hardware removal in the young group (*P* = .015), the revision rate did not differ between the groups (*P* = .483). If the plate arthrodesis is considered in isolation for the type of fixation, in our cohort we found that it performs significantly worse in terms of activity in FFI function final (*P* = .04) and FFI function difference (*P* = .04) compared with screw fixation. In this group, the screw-based arthrodesis appears preferred in terms of functionality. Other possible confounders did not influence outcome significantly in our study. However, we noticed that increased dorsal elevation of the first toe is associated with a worse clinical outcome. A follow-up study on this topic and plate vs screw fixation would certainly be useful.

### Limitations

This is a retrospective single-center study, with a limited number of patients because of the long follow-up. Although we included roughly the same proportion of bilateral cases in both groups, we measured each foot’s outcome separately, which could have been a source of bias. Further limitations include not separately accounting for the impact of various surgical techniques and surgeons or the possibility that other unrecognized comorbid factors could have influenced the functional outcomes in addition to the isolated MTP 1 fusion.

## Conclusion

At an average of 14-year follow-up (minimum 10 years), MTP I arthrodesis in patients under the age of 50 years yielded highly satisfactory results in terms of pain reduction and sustained physical activity. These results further compared favorably both to a matched older cohort of patients aged >60 years and existing literature.

## Supplemental Material

sj-docx-2-fai-10.1177_10711007231205567 – Supplemental material for Ten-Year Minimum Follow-up Study of First Metatarsophalangeal Joint Fusion in Young vs Old Patients Supplemental material, sj-docx-2-fai-10.1177_10711007231205567 for Ten-Year Minimum Follow-up Study of First Metatarsophalangeal Joint Fusion in Young vs Old Patients by Fabrice Scheurer, Stefan M. Zimmermann, Philipp Fischer, Stephan H. Wirth, Silvan Beeler and Arnd F. Viehöfer in Foot & Ankle International

sj-pdf-1-fai-10.1177_10711007231205567 – Supplemental material for Ten-Year Minimum Follow-up Study of First Metatarsophalangeal Joint Fusion in Young vs Old Patients Supplemental material, sj-pdf-1-fai-10.1177_10711007231205567 for Ten-Year Minimum Follow-up Study of First Metatarsophalangeal Joint Fusion in Young vs Old Patients by Fabrice Scheurer, Stefan M. Zimmermann, Philipp Fischer, Stephan H. Wirth, Silvan Beeler and Arnd F. Viehöfer in Foot & Ankle International
