# Supplementary material for: Ten-Year Minimum Follow-up Study of First Metatarsophalangeal Joint Fusion in Young vs Old Patients
Source: Foot Ankle Int. 2023 Dec 30;45(3):217–22. doi: 10.1177/10711007231205567 (PMC10960322; doi:10.1177/10711007231205567)
Supplement: sj-docx-2-fai-10.1177_10711007231205567 – Supplemental material for Ten-Year Minimum Follow-up Study of First Metatarsophalangeal Joint Fusion in Young vs Old Patients [file sj-docx-2-fai-10.1177_10711007231205567.docx]

Supplementary

| Variable | Male | | Screw=0  Plate=1 | | Cheilectomie =1 | | Right side = 1 | | Revision | | Hardware removal | |
| --- | --- | --- | --- | --- | --- | --- | --- | --- | --- | --- | --- | --- |
| **Group** | **A** | B | A | B | A | B | A | B | A | B | A | B |
| VAS_final |  | 0.88 |  | 0.24 |  | 0.68 |  | 0.24 | 0.55 |  | 0.17 |  |
| VAS_diff | 0.95 |  |  | 0.16 | 0.65 |  |  | 0.32 | 0.51 |  | 0.02 |  |
| FFI (pain)_final | 0.94 |  |  | 0.12 | 0.52 |  |  | 0.38 | 1.0 |  | 0.53 |  |
| FFI (pain)_diff |  | 0.92 |  | 0.06 | 0.12 |  |  | 0.40 | 0.85 |  | 0.16 |  |
| FFI (function)_final | 0.56 |  |  | 0.04 | 0.47 |  |  | 0.90 |  | 0.60 | 0.80 |  |
| FFI (function)_diff |  | 0.99 |  | 0.04 | 0.36 |  |  | 0.96 |  | 0.38 | 0.72 |  |
| TAS_final |  | 0.38 | 0.06 |  |  | 0.42 |  | 0.53 | 0.04 |  | 0.91 |  |
| V-TAS_final |  | 0.08 | 0.41 |  |  | 0.33 | 0.79 |  | 0.82 |  |  | 0.18 |
| Table 5 Confounder analysis (Significant differences are marked yellow) | | | | | | | | | | | | |
